# Genetics of Greenbug Resistance in Synthetic Hexaploid Wheat Derived Germplasm

**DOI:** 10.3389/fpls.2019.00782

**Published:** 2019-06-13

**Authors:** Leonardo Crespo-Herrera, Ravi P. Singh, Matthew Reynolds, Julio Huerta-Espino

**Affiliations:** ^1^Centro Internacional de Mejoramiento de Maíz y Trigo (CIMMYT), Global Wheat Program, Mexico, Mexico; ^2^Campo Experimental Valle de Mexico, Instituto Nacional de Investigaciones Forestales, Agrícolas y Pecuarias (INIFAP), Chapingo, Mexico

**Keywords:** wheat, genetics, resistance, greenbug, tolerance, genes

## Abstract

The greenbug, *Schizaphis graminum* Rondani, significantly reduces wheat, *Triticum aestivum* L., grain yields if not controlled. Host plant resistance (HPR) can protect yield, is environmentally friendly and easy to use. Our objectives were to: (1) identify genomic regions associated with *S. graminum* resistance in a recombinant inbred line (RIL) population derived from a cross of “Sokoll” (resistant) and “Weebill1” (moderately susceptible), (2) evaluate Sokoll derived breeding germplasm for resistance, and (3) conduct allelism tests between Sokoll and sources carrying resistance genes *Gba*, *Gbb*, and *Gbd*. Resistance was measured quantitatively and qualitatively using a SPAD meter and visual assessments, respectively. We identified a large effect resistance gene on chromosome arm 7DL of Sokoll, herein referred as *GbSkl*, which contributed up to 24% of the phenotypic variation. Other minor QTL on chromosomes 2B, 3A, and 7B were also identified. The QTL on 2B and 3A originated from Weebill1. Of the Sokoll derived germplasm, 13% displayed resistance. Allelism tests indicated that *GbSkl* could be allelic or tightly linked to the temporarily designated genes *Gba*, *Gbb*, and *Gbd*. Utility of SPAD to determine quantitative variation in resistance phenotyping is demonstrated and breeding efforts are underway to transfer the resistance from Sokoll to new CIMMYT elite germplasm.

## Introduction

Wheat (*Triticum aestivum* L.) is one of the most important cereal crops globally. The production and cultivated area in 2016 were 749 million tons and 220 million hectares, respectively (FAO, 2018). One third (72 million ha) of the total harvested area is accounted by South and Central Asia and North and East Africa (FAO, 2018). These regions contribute to about 25% of the global wheat production with 30% less yield than the global average (FAO, 2018). Major efforts are ongoing worldwide to increase productivity considering climate change and increasing demand scenarios. Wheat production is constrained by abiotic and biotic factors, and climate change can increase the incidence of pests and diseases in addition to heat and drought stresses (Porter et al., 2014).

Among the biotic constraints that severely affect wheat production are aphids; small insects that feed from the phloem sap. The aphid species *Schizaphis graminum* Rondani, commonly known as greenbug, is widely distributed worldwide (Blackman and Eastop, 2007; Leach and Hobbs, 2013; CABI-EPPO, 2018) and can reduce wheat yield by 40–50% especially if infestations occur at early growth stages. Chemical control is widely used, however, smallholder farmers often do not have timely access to insecticides nor have resources, and consequently their crop is more prone to aphid damage. Insecticides are often handled improperly by the smallholder farmers and can have negative effects on non-targeted organisms, including humans (Dewar and Foster, 2017; Mitchell et al., 2017; Woodcock et al., 2017; Bondori et al., 2018).

Host plant resistance (HPR) can significantly contribute to yield protection from aphids and reduce the insecticides usage. Additionally, HPR can reduce production costs thus benefiting farmers more from their crop. This control method, is one of the key component of the integrated pest management, and is the most easily applied method by the farmers, as it is already present in the seeds that they sow and has no additional cost to them.

Host plant resistance to insects is classified in categories that describe the way plants react to insect exposures. Essentially, HPR to insects is composed by three categories: antibiosis, antixenosis and tolerance (Painter, 1941; Kogan and Ortman, 1978; Smith, 2005). Tolerance to aphids is considered more complex than the other categories of resistance, as various plant mechanisms related to allocation patterns and nutrient uptake, antioxidant production, biomass production, photorespiration, photosynthesis, plant growth, and storage capacity are involved (Rosenthal and Kotanen, 1994; Heng-Moss et al., 2003; Boyko et al., 2006; Reddy et al., 2013; Donze-Reiner et al., 2017). Despite the conceptual separation of these categories, it is difficult to isolate them in terms of the actual mechanisms that cause the resistance (Smith and Clement, 2012).

The deployment of insect resistance in elite varieties requires significant investments from the germplasm identification to the development of parental lines and breeding itself. Because phenotypic selection methods are complicated to apply in the field, breeding for insect resistance is a challenging task in large breeding programs that pursue the development of elite germplasm with ample yield gain, stability and resistance to other relevant biotic and abiotic factors. Nonetheless, marker assisted breeding (MAB) can be an efficient tool to utilize in breeding programs to transfer and select resistance to aphids. The successful implementation of MAB lies in the identification of molecular markers closely linked to resistance genes, which is achieved through genetic analyses, linkage mapping (QTL analysis), or linkage disequilibrium related methods.

Previous studies have identified genes (*Gb2* and *Gb6*) on chromosome 1R from rye (Puterka and Peters, 1989; Lu et al., 2010) and in *Aegilops speltoides* Tausch (*Gb5*) (Tyler et al., 1987; Dubcovsky et al., 1998; Crespo-Herrera et al., 2013). Synthetic hexaploid wheats (SHWs) are other highly valuable source of resistance to *S. graminum* (Lage et al., 2003). The SHWs are the product of the human-induced hybridization between tetraploid species carrying the “A” and “B” genomes of wheat, with accessions of the “D” genome ancestor goat grass (*Aegilops tauschii* Coss). SHWs can be directly crossable with the adapted germplasm, hence transfer of desirable traits from wild relatives is possible. Studies have identified resistance genes on chromosome 7DL of *A. tauschii*, i.e., *Gb3*, *Gb4*, *Gb7*, *Gba*, *Gbb*, *Gbc*, *Gbd*, *Gbx*, and *Gbz*, and it has been demonstrated that these are single dominant genes (Puterka and Peters, 1989; Zhu et al., 2005; Crespo-Herrera et al., 2014; Tan et al., 2017). However, the relationships between the SHW identified genes need to be established.

The bread-wheat breeding program at the International Maize and Wheat Improvement Centre (CIMMYT) has recently undertaken the task of transferring resistance to aphids (*S. graminum* and *Rhopalosiphum padi* L.) to elite breeding germplasm. As part of this endeavor, CIMMYT germplasm was screened for resistance against these pests, which allowed the identification of the drought tolerant line “Sokoll” (Reynolds et al., 2007) also resistant to *S. graminum*. Sokoll is a synthetic hexaploid derived line, pedigree: “PASTOR/3/ALTAR 84/*A. squarrosa* (TAUS)//OPATA,” and has been used in CIMMYT’s wheat breeding program to develop drought tolerant germplasm.

The objectives of our study were: (1) to identify the genomic regions associated with resistance to *S. graminum* in the CIMMYT’s synthetic hexaploid derived wheat Sokoll; (2) characterize the marker-*S. graminum* resistance association in a set of Sokoll derived lines; and (3) determine the association of the resistance between Sokoll and *Gba*, *Gbb*, and *Gbd S. graminum* resistance genes of SHW origin through allelism tests, since the *A. squarrosa* (*A. tauschii*) accession in Sokoll is unknown.

## Materials and Methods

### Plant Materials

Three sets of germplasm, developed at the International Maize and Wheat Improvement Centre (CIMMYT), were evaluated: (1) 228 F_6_ recombinant inbred lines (RILs) derived from the cross of the SHW derived line Sokoll (*S. graminum* resistant) with Weebill1 (*S. graminum* moderately susceptible), (2) A set of 46 Sokoll derived breeding lines, and (3) Four F_2_ derived F_3_ populations of 94, 92, 98, and 98 families from the crosses between Sokoll and the SHWs previously reported to carry *S. graminum* resistance genes: “Ceta/A. squarrosa (1027),” “Seri//T. dicoccon PI94623/A. Squarrosa (1027),” “Croc_1/A. Squarrosa (224)”, and “Altar 84/A. Squarrosa (328)” (Lage et al., 2003; Zhu et al., 2005; Crespo-Herrera et al., 2014).

### Aphid Population

Clones of *S. graminum* were collected from wheat fields at CIMMYT’s experimental station located in Ciudad Obregon, Sonora, Mexico (27° 37’ N, 109° 93’ W). Aphids were reared on the CIMMYT’s bread wheat line “Reedling #1” under controlled conditions at 24 ± 2°C and 16:8 (light:dark) photoperiod with high-pressure sodium lamps as light source.

After the establishment of the aphid cultures, the *S. graminum* the virulence/avirulence pattern was determined by evaluating a set of differential lines, the parents of the RILs and the F_2_:F_3_ families (Table 4). The virulence/avirulence pattern of the aphid population was similar to the biotype I in the United States (Porter et al., 1997).

### Phenotyping and Data Analysis

The plant materials were evaluated at seedling stage. Five seeds of each of the Sokoll/Weebill1 RILs were planted as tufts in flats of 40 cm × 53 cm × 7 cm. The evaluations consisted of two treatments, infested vs. non-infested, arranged in augmented incomplete blocks where the resistant (Sokoll) and susceptible (Weebill1) progenitors of the RILs were replicated 8 and 6 times in each flat as controls, respectively. After emergence, each tuft was carefully thinned to allow the growth of one seedling of similar size for both treatments. The flats were grown side by side in growth chambers with controlled light (16:8) and temperature (22 ± 2°C).

Each seedling of the infested treatment was exposed to 15–20 individuals of *S. graminum* at the 2nd–3rd leaf stage. The infestations were conducted every second day during 10–12 days to ensure high and homogeneous aphid pressure within flats. Since the flats were grown side by side, the non-infested flats were treated with a 0.2% solution of Admire^®^ (Bayer, Imidacloprid @ 30.2%) 1 day before the infestation treatment was applied, with the objective to avoid aphid establishment and movement from the infested flats. This insecticide is a commercially available product in Mexico, and its active ingredient has been tested to assess the resistance to aphids in wheat (Dunn et al., 2007).

Chlorophyll content was measured on each seedling with a SPAD meter (Minolta^®^). Three readings along the first fully developed leaves were taken and averaged. In addition, a visual assessment of the aphid damage on a 0–100 scale was also recorded, the scale was based on the percentage of chlorosis displayed by the plants under aphid feeding. Data were collected when the susceptible check displayed 60–70% damage (chlorosis). The evaluation of RILs was repeated three times, i.e., once a full round of evaluations was finished with the augmented design, the RILs were evaluated two more times.

A similar procedure was followed for the Sokoll derived lines and the F_2_:F_3_ populations to conduct the allelism tests, except that the tufts were not thinned, and the non-infested treatment was not established, hence we evaluated between 460 and 490 total seedlings per populations. The susceptible check for this evaluations was “Pavon F76”, a hard-white spring wheat variety highly susceptible to aphids (Crespo-Herrera et al., 2013) and the resistant check was Sokoll. Only the qualitative scores (S, susceptible; R, resistant) were given based on the seedling response to the aphid feeding.

The classification of each of the F_2_:F_3_ families were used to assess the hypothesis of 15:1 (Resistant:Susceptible) ratio for two independent dominant genes in a *Chi*-squared test, given that each family is expected to segregate in that ratio for two independent genes. The families displaying any sign of damage, or abnormal observations were re-evaluated to confirm the results by exposing each family to aphid feeding. For this, Twenty-five seeds of each family and the susceptible check were planted in rows on the flats previously described, and each row of seedlings were infested following the same procedures as described above.

Phenotypic data were analyzed with the package lme4 (Bates et al., 2015) in the R software v.3.4 (R Development Core Team, 2013). Best linear unbiased estimators (BLUEs) were obtained from the analysis of the RILs by fitting the lines and treatments as fixed effects, whereas the incomplete blocks and the experiment repetitions where considered as random effects. We obtained heritability estimates for the mapping population from the variance components by fitting the RILs as random effects, but keeping the parental lines and treatments as fixed effects in the linear mixed model.

### Genotyping

The Sokoll/Weebill1 RILs and the Sokoll derived lines were genotyped with the Diversity Array Technology (DArT), and DArT-Seq at the laboratory of Genetic Analysis Service for Agriculture in CIMMYT, Mexico. The DArT-Seq procedure consists on digestion, primer and barcode ligation, amplification and sequencing processes of the DNA samples (Sansaloni et al., 2011). Unlike DArT-Seq, the array procedure (DArT) lacks the sequencing process and yields a presence/absence pattern of the markers by hybridizing the probes to the wheat array (Wenzl et al., 2004; Akbari et al., 2006). In total there were 95,958 markers processed prior to the linkage analysis. Markers not considered for further analysis were those with more than 20% missing data, minor allele frequency lower than 5% and those that were monomorphic between the parents of the RILs.

### Linkage and QTL Analysis

Linkage and QTL analyses were performed for the Sokoll/Weebill1 RIL population. Markers were first grouped with the ICIMapping software (Meng et al., 2015) using the DArT chromosome locations as anchoring information and a high LOD threshold (30.0) for grouping unanchored markers. Subsequently, each group was ordered using the ASMap package (Taylor and Butler, 2017), which contains the linkage map functions of the MSTMap (Wu et al., 2008) for R language. The program takes the minimum spanning tree of a graph for grouping and ordering. The utilization of these two tools allowed us the use of the prior knowledge of the markers (ICIMapping) and the ordering efficiency of ASMap.

The QTL analysis was conducted with the R package R/qtl (Broman and Sen, 2009) to identify genomic regions associated with chlorophyll content, chlorophyll loss and aphid damage. First, interval mapping was implemented with the Haley-Knott regression method (Haley and Knott, 1992). Significant genomic regions were identified after a 1,000 permutations run at a threshold of the 5% tail of the null distribution. Significant main effect QTL were then further examined in a multiple interval mapping framework to refine positions, estimate QTL effects and the proportion of variance explained by the QTL and the QTL model.

## Results

### Phenotypic Analysis of Recombinant Inbred Lines (RILs)

In the infested treatment, seedlings of the parental lines, Sokoll (resistant) and Weebill1 (susceptible), displayed 28.3 and 11.5 units of chlorophyll content (SPAD units), respectively in contrast to 31.3 (Sokoll) and 29.1 (Weebill1) units for their non-infested treatments, which resulted in chlorophyll losses of 9.4 and 60.3% on average, respectively for the two parents. The visual damage score for Sokoll and Weebill1 was 1 and 68.4% on average, respectively. The analysis of the phenotypic data indicated a significant variation between RILs for all recorded traits. The damage score ranged from 3.5 to 84.3% (*F*_229,392.9_ = 1.24, *P* < 0.0001), and the heritability (*h*^2^) was 0.52. The chlorophyll content of the RILs in the infested treatment, ranged from 4.6 to 35.2 (*F*_229,615_ = 11.72, *P* < 0.0001) and *h*^2^ = 0.89, whereas in the non-infested treatment it ranged from 23.3 to 39.4 (*F*_229,627_ = 2.07, *P* < 0.0001) and *h*^2^ = 0.49. The chlorophyll loss, expressed as percentage of SPAD units and as a function of chlorophyll content between infested vs. non-infested treatments, ranged from −24.46 to 84.15%.

**FIGURE 1:**
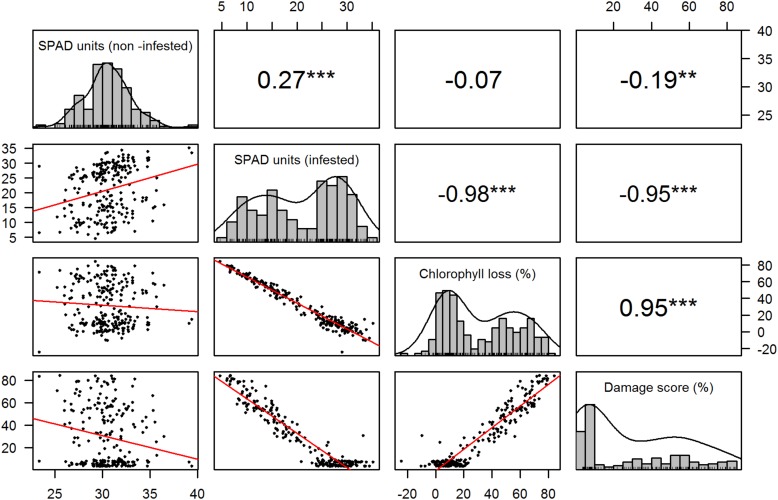
Trait distribution and correlation. The diagonal displays the histogram for each recorded trait. The panels below and above the diagonal represent the scatter plots and correlation values between traits, respectively. ^∗∗^*p* = 0.01, ^∗∗∗^*p* < 0.001.

**FIGURE 2:**
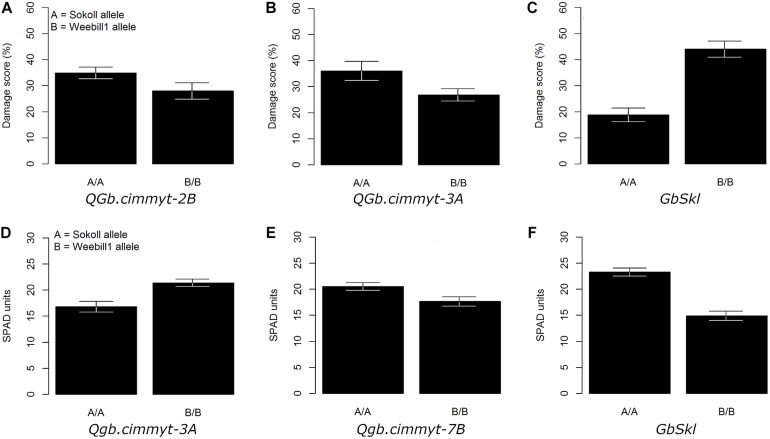
Adjusted mean trait (±SE) of main QTL effects across Sokoll/‘Weebill1 RILs. **(A–C)** Correspond to damage score of QTL on 2B, 3A, and *GbSkl* in 7DL, respectively. **(D–F)** Correspond to chlorophyll content (SPAD units) of QTL on 3A, 7B, and *GbSkl*, respectively.

The correlation analysis between recorded traits indicated a significantly positive (*r* = 0.27, *p* < 0.001) association between the chlorophyll content of the RILs in both treatments, but an insignificant close to zero (*r* = −0.07, *p* = 0.9) correlation between chlorophyll loss and chlorophyll content in the non-infested treatment (Figure 1). High and significant (*r* > |0.9|, *p* < 0.001) correlations were detected between the traits related to the infested treatment (Figure 2).

The histograms of chlorophyll content, loss and damage scores, displayed a significant departure (*D* = 0.04, *p* < 0.005) from a unimodal distribution according to the Hartigan’s dip test for unimodality. The Sarle’s bimodality coefficient of the recorded traits in the infested treatments was *b* > 0.6, which suggests a bimodal distribution, which for genetic interpretations indicates the presence of a gene with a major effect segregating in the RIL population.

### Linkage and QTL Analysis

We used 5,259 DNA markers for QTL analysis after conducting the quality control of the genotypic data. From these, 24 linkage groups (LGs) were built, one for each wheat chromosome plus additional LGs for each of the chromosomes 3B, 5B, and 6B. The number of markers per LG ranged from 20 (4D) to 650 (7B). The size of the LGs spanned from 33.34 cM (4D) to 597.13 cM (6A), and the total distance of the LG summed to 6,507.03 cM. On average, the LG were comprised by one marker every 1.24 cM, but this value ranged for individual LG from 0.29 cM (1B) to 5.78 cM (5D). The markers were distributed across the three wheat genomes as follows: 32.7, 55.1, and 12.2%, for the A, B, and D genomes, respectively.

**FIGURE 3:**
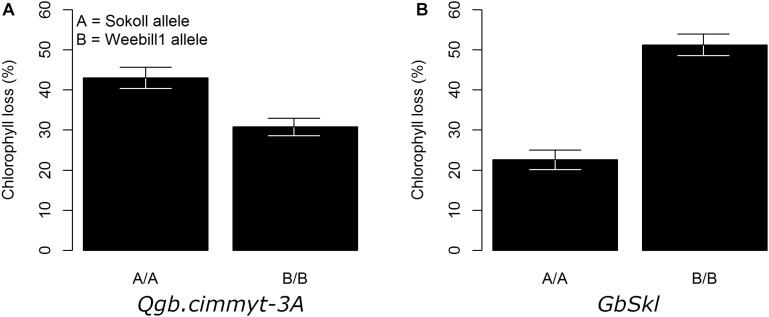
Adjusted mean trait (±SE) of chlorophyll loss for main QTL effect across Sokoll/Weebill1 RILs. **(A)** Chlorophyll loss for QTL on 3A. **(B)** Chlorophyll loss for *GbSkl* on 7DL.

**FIGURE 4:**
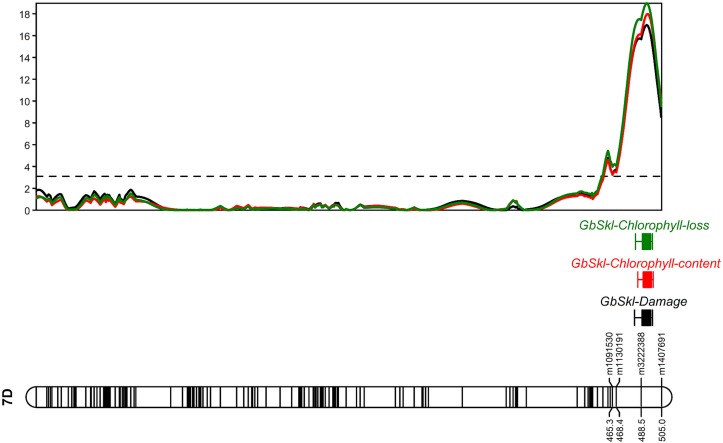
LOD profile of damage score, chlorophyll content and loss on linkage group (LG) assigned to chromosome 7D.

The QTL models explained 37.8, 40.2, and 37.1% of the total phenotypic variation for damage score, chlorophyll content and chlorophyll loss, respectively (Table 1). For all recorded traits, the QTL analysis of the Sokoll/Weebill1 RILs indicated the presence of a genomic region with large effect (Table 1 and Figures 2C,F, 3B) on chromosome 7DL (Figure 4) of Sokoll that explained between 16.5 and 24.1% of the phenotypic variation (Table 1). This locus is herein referred as *GbSkl*. The closest marker to *GbSkl* was m3222388 (Figure 4). An additional QTL (*QGb.cimmyt-3A*) was also found for all traits on chromosome 3A of Weebill1 that explained a lesser amount of phenotypic variation, up to 5.4% (Table 1 and Figures 2B,D, 3A). Other minor QTL were found on chromosomes 2B (*QGb.cimmyt-2B*) for damage score, which accounted for a reduced amount of phenotypic variation (Table 1 and Figure 2A), and another on chromosome 7B (*QGb.cimmyt-7B*) for chlorophyll content which explained 3.9% of the phenotypic variation (Table 1 and Figure 2E). The closest marker to these QTL were m3951322 and m4439909, respectively.

**TABLE 1:** Quantitative trait loci (QTL) for *S. graminum* resistance identified in the Sokoll/Weebill1 mapping population.

				**Position**	**Interval**				**PVE QTL**
**Trait**	**QTL**	**Chromosome**	**Flanking markers**	**(cM)**	**(cM)^†^**	**LOD**	**Effect**	**PVE^‡^**	**model**
Damage	*QGb.cimmyt-2B*	2B	m39513221; m3951322^*^	122.1	117–131	4.1	–3.1	1.7	37.8
	*QGb.cimmyt-3A*	3A	m3385293; m1070254	94	88–106	7.2	–7.4	5.3	
	*GbSkl*	7DL	m3222388^*^; m1407691	493	484–498	15.9	14.8	20.1	
Chlorophyll content	*QGb.cimmyt-3A*	3A	m3385293; m1070254^*^	93	84–105	5.1	2.1	3.8	40.2
	*QGb.cimmyt-7B*	7B	m999736; m4439909^*^	367	362–370	4.1	–1.9	3.9	
	*GbSkl*	7DL	m3222388^*^; m1407691	493	486–498	12.5	–4.4	16.5	
Chlorophyll loss	*QGb.cimmyt-3A*	3A	m3949605; m4404564^*^	99.8	91–110	6.8	–6.5	5.4	37.1
	*GbSkl*	7DL	m3222388^*^; m1407691	492	486–497	18.9	15.3	24.1	

^†^1.5 LOD drop confidence interval of the QTL peak; ^‡^Percentage of variance explained by the QTL; ^*^Closest marker to QTL.

### Sokoll Derived Lines

Among the Sokoll derived lines evaluated, 13% exhibited a resistant phenotypic reaction (Table 2). The full list of lines with their pedigree, marker and phenotypic response is given in the Supplementary Table S1.

**TABLE 2:** Marker response of Sokoll derived lines resistant to *S. graminum* damage.

	**Chromosome**	**3A**		**7D**		**Response**
**GID**	**Pedigree**	**m3385293**	**m1070254**	**m3222388**	**m1407691**	
3825355	Sokoll	A	A	A	A	R
2448314	Weebill1	B	B	B	B	S
6001179	Sokoll//FRTL/2^*^PIFED	A	A	A	B	R
6001642	Sokoll^*^2/4/CHEN/AEGILOPS SQUARROSA (TAUS)//FCT/3/STAR	A	A	B	B	R
6692338	Sokoll/3/PASTOR//HXL7573/2^*^BAU/4/Sokoll/WBLL1	B	A	A	A	R
6933732	TILHI/Sokoll^*^2//KINGBIRD #1	A	B	A	A	R
6939941	W15.92/4/PASTOR//HXL7573/2^*^BAU/3/WBLL1/5/Sokoll/3/PASTOR//HXL7573/2^*^BAU	B	A	A	A	R
7178649	BAVIS #1^*^2/4/PASTOR//HXL7573/2^*^BAU/3/Sokoll/WBLL1	A	B	A	A	R

For marker m3222388, linked to *GbSkl*, 2.1% lines displayed a false negative response, while 19.5% showed false positive responses (Supplementary Table S1). For marker m1407691, also flanking the 7DL QTL, there were 4.3% false negatives and 10.8% false positives. The combined response of these markers had 8.7% false positive and 2.1% false negative rates.

For the QTL on 3A the flanking markers displayed a 4.3% false negative responses, and 15.2 and 45.7% false positive responses for markers m3385293 and m1070254, respectively. The combined response of these two markers had 13% false positives, however; none had a false negative response.

### Allelism Tests

We did not observe any susceptible seedlings or segregating families in the F_2_:F_3_ populations derived between the crosses of Sokoll and *Gba*, *Gbb*, and *Gbd* sources (Table 3). Hence, with the Chi-squared test the null hypothesis of 15:1 (Resistant:Susceptible) phenotypic segregation ratio was rejected for the *GbSkl* and *Gba*, *Gbb*, and *Gbd*, indicating that the presence of an allelic gene or tightly linked genes could be involved in the resistance to *S. graminum*.

**TABLE 3:** Phenotypic segregation patterns of F_2_ derived F_3_ populations developed for allelism tests.

		**Number**						
**Population**	**Gene**	**of lines**	**O-R^a^**	**O-S^b^**	**E-R^c^**	**E-S^d^**	**χ^2^-value^e^**	***p*^f^**
Sokoll//Ceta/*A. squarrosa* (1027)	*Gba*	94	94	0	88.12	5.87	6.26	0.012
Sokoll/3/Seri//*T. dicoccon* PI94623/A. Squarrosa (1027)	*Gba*	92	92	0	86.25	5,75	6.04	0.013
Sokoll//Croc_1/*A. squarrosa* (224)	*Gbb*	98	98	0	91.87	6.12	6.98	0.011
Sokoll//Altar 84/*A. squarrosa* (328)	*Gbd*	98	98	0	91.87	6.12	6.98	0.011

*${}^{a}$Observed Resistant; ${}^{b}$Observed Susceptible; ${}^{c}$Expected Resistant; ${}^{d}$Expected Susceptible; ${}^{e}$Calculated *Chi*-squared value for the null hypothesis of the populations to segregate 15:1 (R:S); ${}^{f}$Probability of deviation from expectations.*

**TABLE 4:** Response of differential lines to *S. graminum* feeding for biotype determination.

GID^*^	**Cross/Pedigree**	**Gene**	**Response**
256679	DICKINSON 28A	*Gb1*	S
154175	TAM 107	*Gb2*	S
39992	TAM 200	*Gb2*	S
8198455	LARGO	*Gb3*	R
8198456	KS82H1640GB	*Gb4*	R
7615722	Pavon 76, 20” + 7A.7S	*Gb5*	R
8198457	GRS1201	*Gb6*	R
8198458	KS89WGRC4	*Gbx1*	R
2448314	Weebill1	–	S
3825355	Sokoll	–	R
174364	Ceta/*A. squarrosa* (1027)	*Gba*	R
6581138	Seri//*T. dicoccon* PI94623/	*Gba*	R
	*A. squarrosa* (1027)		
180015	Croc_1/*A. squarrosa* (224)	*Gbb*	R
357971	Altar 84/*A. squarrosa* (328)	*Gbd*	R

^*^GID, Germplasm identification number.

## Discussion

Incorporating HPR to insects in elite germplasm that can potentially become commercial varieties is of great value due to environmental considerations associated with reduced insecticide use, as well as benefits to non-targeted organisms including human, and reduction of production costs and health risks to farmers. The accurate identification and clear understanding of the genetics of resistance are important for breeding programs to efficiently incorporate HPR to insects as a selection trait, either directly (phenotypic selection) and/or indirectly (MAB).

The analysis of the phenotypic data supports the conclusion that a major gene controls the resistance to *S. graminum* in Sokoll, as the population could be classified in two groups but with phenotypic variation within the groups according to the bimodality parameters.

The observed correlation patterns of the phenotypic data demonstrate that the chlorophyll content in non-infested treatments is not indicative of resistance to *S. graminum* (Figure 1). The low but significant correlation of chlorophyll content between the evaluated treatments indicated that plants with higher chlorophyll content tended to also have higher chlorophyll content when exposed to aphids, but not to the level of exhibiting resistance, which is supported by the null association between chlorophyll loss and content in the non-infested treatment (Figure 1). Furthermore, given the high correlation exhibited between chlorophyll content, chlorophyll loss and damage score, despite the null association between chlorophyll loss and chlorophyll content in non-infested plants, it is possible to conclude that, taking SPAD measurements in infested treatments may be sufficient as a rapid evaluation of resistance, as long as appropriate controls are included in the experimental design. Another important aspect to note for phenotyping purposes is that the visual score was highly correlated with chlorophyll content and loss, indicating that this alone can be a good parameter to assess tolerance in mapping studies, as the same important genomic regions were detected using the SPAD meter related traits (Table 1).

Our study aimed to evaluate quantitatively the ability of Sokoll/Weebill1 RILs and additional Sokoll derived material to withstand *S. graminum* damage (tolerance) under high aphid pressure in seedling experiments, and map the genomic regions conferring resistance in Sokoll. Typically, tolerance to *S. graminum* is evaluated qualitatively for gene mapping studies (Weng and Lazar, 2002; Zhu et al., 2004, 2005; Weng et al., 2005). Although SPAD measurements had been used for evaluating tolerance (Lage et al., 2003; Boina et al., 2005), it had not been used as a trait for genetic studies.

Our screening methodology allowed us to identify genomic regions with small effects that are associated with *S. graminum* tolerance, in addition to a region harboring the major effect resistance gene *GbSkl* on chromosome 7DL (Weng and Lazar, 2002; Boyko et al., 2004; Zhu et al., 2005). Interestingly, the *QGb.cimmyt-2B* and *QGb.cimmyt-3A* alleles from Weebill1 contributed small effects to the phenotypic variation of the evaluated traits (Figures 2A,B,D, 3A). Such a small effects alone are not enough for the lines to display acceptable levels of protection, however, in combination with *GbSkl* they can play an important role in conferring higher levels of resistance. It is relevant to note that these QTL with small effects were located on the “A” and “B” genomes of wheat, unlike most of the reported genes on the “D” genome from *A. tauschii*. Another, reported gene in wheat, but not in the “D” genome is *Gby* on chromosome 7A, which confers tolerance and antibiosis (Boyko et al., 2004; Boina et al., 2005).

Sokoll has been used as one of the parents for the development of CIMMYT’s drought tolerant germplasm. Even though, DArT-Seq markers cannot be readily used for MAB and require further efforts to be transformed in user friendly assays, the results from the marker response highlight the importance of having flanking markers to reduce the false positive rate for MAB purposes. Furthermore, the recombinant lines can be used for fine mapping and developing molecular markers that are in tighter linkage to the resistance gene (Table 2). Efforts are underway to characterize a larger set of Sokoll derivatives in CIMMYT breeding germplasm to determine the phenotypic and marker responses using KASP assays we developed based on additional studies we conducted (Crespo-Herrera et al., 2014).

The sequence of the DArT-seq markers flanking *GbSkl* were investigated in the reference wheat genome sequence (IWGSC RefSeq v1.0) in the Ensembl database (Zerbino et al., 2018), these markers (m3222388 and m1407691) were located 5.7 Mbp apart on 7DL between the physical position of 593.4 and 599.1 Mbp, a region that encloses 56 genes predicted to code for 35 protein superfamilies. The superfamilies include: ricin B-like lectins, terpenoid cyclases/protein prenyltransferases, terpenoid synthases, serine metabolism enzymes, cysteine proteinases, and leucine rich repeats, which can be related to plant defenses to aphids (Goggin, 2007; Smith and Boyko, 2007).

The results from the allelism tests suggest that the major gene in Sokoll, *GbSkl*, could be allelic or in tight linkage with *Gba*, *Gbb*, and *Gbd* reported genes, as the F_2_:F_3_ populations did not segregate for *S. graminum* resistance (Table 3). Additional testing is required to determine the allelic relations between these genes and other sources, such as *Gb7*, *Gbc*, and *Gbz*. Due to a lack of seed availability we could not develop these additional populations. Derived from the pedigree information, it is possible that *GbSkl* is related to *Gb7*, however, this cannot be confirmed by pedigree alone as the *A. tauschii* accession that is the source of *Gb7* appears to be different from *GbSkl* (Tan et al., 2017). There is reported evidence that *Gbd* is different from *Gbz* (Zhu et al., 2005), and also that *Gbz* is allelic or tightly linked to *Gb3* (Zhu et al., 2004). Therefore, it could be inferred that *Gba*, *Gbb*, *Gbd* and *GbSkl* are different from *Gb3*. However, more work is required to determine the relationship between all these *S. graminum* resistance genes. A definite way to fully deciphering it is by cloning the genes involved in the resistance.

Our results contribute to unraveling the genetic relationships between certain *S. graminum* resistance genes, and in the identification of key genomic regions that contribute to the phenotypic variation for *S. graminum* resistance in Sokoll wheat. Our results also suggest that measuring chlorophyll content with a SPAD meter in *S. graminum* infested plants can be a quick and standardized evaluation method for determining resistance. Breeding efforts are undergoing to transfer the resistance to newer elite germplasm and the resistant materials being distributed to CIMMYT collaborators for evaluation in local environments where this aphid is a concern.

## Data Availability

The raw data supporting the conclusions of this manuscript will be made available by the authors, without undue reservation, to any qualified researcher.

## Author Contributions

LC-H established the screening methodology, conducted the phenotyping, data analysis, and wrote the main manuscript. LC-H, JH-E, and RS conducted the data analysis, developed the parental lines of RILs, and the allelism test populations. MR developed the RILs. All authors reviewed and approved the final version of the manuscript.

## Conflict of Interest Statement

The authors declare that the research was conducted in the absence of any commercial or financial relationships that could be construed as a potential conflict of interest.

## Supplementary Material

TABLE S1 Marker *S. graminum* response of selected Sokoll derived germplasm.

## References

[B1] Akbari M. Wenzl P. Caig V. Carling J. Xia L. Yang S. (2006). Diversity arrays technology (DArT) for high-throughput profiling of the hexaploid wheat genome. *Theor. Appl. Genet.* 113 1409–1420. 10.1007/s00122-006-0365-4 17033786

[B2] Bates D. Mächler M. Bolker B. Walker S. (2015). Fitting linear mixed-effects models using lme4. *J. Stat. Softw.* 67 1–48. 10.18637/jss.v067.i01

[B3] Blackman R. L. Eastop V. F. (2007). “Taxonomic issues,” in *Aphids as Crop Pests.* eds Van Emden H. F. Harrington R. (Wallingford: CAB International), 1–29.

[B4] Boina D. Prabhakar S. Smith C. M. Starkey S. Zhu L. Boyko E. (2005). Categories of resistance to biotype I greenbugs (Homoptera: Aphididae) in wheat lines containing the greenbug resistance genes *Gbx* and *Gby*. *J. Kansas Entomol. Soc.* 78 252–260.

[B5] Bondori A. Bagheri A. Damalas C. A. Allahyari M. S. (2018). Use of personal protective equipment towards pesticide exposure: farmers’ attitudes and determinants of behavior. *Sci. Total Environ.* 639 1156–1163. 10.1016/J.SCITOTENV.2018.05.203 29929284

[B6] Boyko E. Starkey S. Smith M. (2004). Molecular genetic mapping of *Gby*, a new greenbug resistance gene in bread wheat. *Theor. Appl. Genet.* 109 1230–1236. 10.1007/s00122-004-1729-2 15309299

[B7] Boyko E. V. Smith C. M. Thara V. K. Bruno J. M. Deng Y. Starkey S. R. (2006). Molecular basis of plant gene expression during aphid invasion: wheat Pto-and Pti-like sequences are involved in interactions between wheat and Russian wheat aphid (Homoptera: Aphididae). *J. Econ. Entomol.* 99 1430–1445. 16937702 10.1603/0022-0493-99.4.1430

[B8] Broman K. W. Sen Ś. (2009). *R/qtl: QTL Mapping in Experimental crosses.* eds Gail M. Krickeberg K. Samet J. Tsiatis A. Wong W. New York, NY: Springer Science+Business Media.

[B9] CABI-EPPO (2018). *Plantwise Knowledge Bank.* Wallingford: CABI.

[B10] Crespo-Herrera L. A. Akhunov E. Garkava-Gustavsson L. Jordan K. W. Smith C. M. Singh R. P. (2014). Mapping resistance to the bird cherry-oat aphid and the greenbug in wheat using sequence-based genotyping. *Theor. Appl. Genet.* 127 1963–1973. 10.1007/s00122-014-2352-5 25112202

[B11] Crespo-Herrera L. A. Smith C. M. Singh R. P. Åhman I. (2013). Resistance to multiple cereal aphids in wheat–alien substitution and translocation lines. *Arthropod. Plant. Interact.* 7 535–545. 10.1007/s11829-013-9267-y

[B12] Dewar A. Foster S. (2017). Overuse of pyrethroids may be implicated in the recent BYDV epidemics in cereals. *Outlooks Pest Manag.* 28 7–12. 10.1564/v28_feb_03

[B13] Donze-Reiner T. Palmer N. A. Scully E. D. Prochaska T. J. Koch K. G. Heng-Moss T. (2017). Transcriptional analysis of defense mechanisms in upland tetraploid switchgrass to greenbugs. *BMC Plant Biol.* 17:46. 10.1186/s12870-017-0998-2 28209137 PMC5314684

[B14] Dubcovsky J. Lukaszewski A. J. Echaide M. Antonelli E. F. Porter D. R. (1998). Molecular characterization of two *Triticum speltoides* interstitial translocations carrying leaf rust and greenbug resistance genes. *Crop Sci.* 38 1655–1660.

[B15] Dunn B. L. Carver B. F. Baker C. A. Porter D. R. (2007). Rapid phenotypic assessment of bird cherry-oat aphid resistance in winter wheat. *Plant Breed.* 126 240–243. 10.1111/j.1439-0523.2007.01345.x

[B16] FAO (2018). *FAOSTAT Database.* Available at: http://faostat.fao.org/ (accessed July 8, 2018)

[B17] Goggin F. L. (2007). Plant-aphid interactions: molecular and ecological perspectives. *Curr. Opin. Plant Biol.* 10 399–408. 10.1016/j.pbi.2007.06.004 17652010

[B18] Haley C. S. Knott S. A. (1992). A simple regression method for mapping quantitative trait loci in line crosses using flanking markers. *Heredity* 69 315–324. 16718932 10.1038/hdy.1992.131

[B19] Heng-Moss T. M. Ni X. Macedo T. Markwell J. P. Baxendale F. P. Quisenberry S. S. (2003). Comparison of chlorophyll and carotenoid concentrations among Russian wheat aphid (Homoptera: Aphididae)-infested wheat isolines. *J. Econ. Entomol.* 96 475–481. 14994818 10.1093/jee/96.2.475

[B20] Kogan M. Ortman E. F. (1978). Antixenosis- a new term proposed to define Painter’s “non-preference” modality of resistance. *Bull. Entomol. Soc. Am.* 24 175–176.

[B21] Lage J. Skovmand B. Andersen S. B. (2003). Characterization of greenbug (Homoptera: Aphididae) resistance in synthetic hexaploid wheats. *J. Econ. Entomol.* 96 1922–1928. 14977134 10.1093/jee/96.6.1922

[B22] Leach M. C. Hobbs S. L. A. (2013). Plantwise knowledge bank: delivering plant health information to developing country users. *Learn. Publ.* 26 180–185. 10.1087/20130305

[B23] Lu H. Rudd J. C. Burd J. D. Weng Y. (2010). Molecular mapping of greenbug resistance genes *Gb2* and *Gb6* in T1AL.1RS wheat-rye translocations. *Plant Breed.* 129 472–476. 10.1111/j.1439-0523.2009.01722.x

[B24] Meng L. Li H. Zhang L. Wang J. (2015). QTL IciMapping: integrated software for genetic linkage map construction and quantitative trait locus mapping in biparental populations. *Crop J.* 3 269–283. 10.1016/j.cj.2015.01.001

[B25] Mitchell E. A. D. Mulhauser B. Mulot M. Mutabazi A. Glauser G. Aebi A. (2017). A worldwide survey of neonicotinoids in honey. *Science* 358 109–111. 10.1126/science.aan3684 28983052

[B26] Painter R. H. (1941). The economic value and biologic significance of insect resistance in plants. *J. Econ. Entomol.* 34 358–367.

[B27] Porter D. R. Burd J. D. Shufran K. A. Webster J. A. Teetes G. L. (1997). Greenbug (Homoptera: Aphididae) biotypes: selected by resistant cultivars or preadapted opportunists? *J. Econ. Entomol.* 90 1055–1065.

[B28] Porter J. R. Xie L. Challinor A. J. Cochrane K. Howden S. M. Iqbal M. M. (2014). “Food security and food production systems,” in *Climate Change 2014: Impacts, Adaptation, and Vulnerability. Part A: Global and Sectoral Aspects. Contribution of Working Group II to the Fifth Assessment Report of the Intergovernmental Panel on Climate Change.* eds Field C. B. Barros V. R. Dokken D. J. Mach K. J. Mastrandrea M. D. Bilir T. E. (New York, NY: Cambridge University Press), 485–533.

[B29] Puterka G. J. Peters D. C. (1989). Inheritance of greenbug, *Schizaphis graminum* (rondani), virulence to *Gb2* and *Gb3* resistance genes in wheat. *Genome* 32 109–114.

[B30] R Development Core Team (2013). *R: a Language and Environment for Statistical Computing.* Vienna: R Foundation of Statistical. Computing. 409.

[B31] Reddy S. K. Weng Y. Rudd J. C. Akhunova A. Liu S. (2013). Transcriptomics of induced defense responses to greenbug aphid feeding in near isogenic wheat lines. *Plant Sci.* 212 26–36. 10.1016/j.plantsci.2013.08.002 24094051

[B32] Reynolds M. Dreccer F. Trethowan R. (2007). Drought-adaptive traits derived from wheat wild relatives and landraces. *J. Exp. Bot.* 58 177–186. 10.1093/jxb/erl250 17185737

[B33] Rosenthal J. P. Kotanen P. M. (1994). Terrestrial plant tolerance to herbivory. *Trends Ecol. Evol.* 9 145–148. 10.1016/0169-5347(94)90180-5 21236799

[B34] Sansaloni C. Petroli C. Jaccoud D. Carlilng J. Detering F. Grattapaglia D. (2011). “Diversity arrays technology (DArT) and next-generation sequencing combined: genome-wide, high throughput, highly informative genotyping for molecular breeding of Eucalyptus,” in *Proceedings of the IUFRO Tree Biotechnology Conference 2011: from Genomes to Integration and Delivery.* ed. Grattpaglia D. (Arraial d’Ajuda: BioMed Central).

[B35] Smith C. M. (2005). *Plant Resistance to Arthropods: Molecular and Conventional Approaches.* Dordrecht: Springer.

[B36] Smith C. M. Boyko E. V. (2007). The molecular bases of plant resistance and defense responses to aphid feeding: current status. *Entomol. Exp. Appl.* 122 1–16. 10.1111/j.1570-7458.2006.00503.x

[B37] Smith C. M. Clement S. L. (2012). Molecular bases of plant resistance to arthropods. *Annu. Rev. Entomol.* 57 309–328. 10.1146/annurev-ento-120710-100642 21910639

[B38] Tan C.-T. Yu H. Yang Y. Xu X. Chen M. Rudd J. C. (2017). Development and validation of KASP markers for the greenbug resistance gene *Gb7* and the Hessian fly resistance gene *H32* in wheat. *Theor. Appl. Genet.* 130 1867–1884. 10.1007/s00122-017-2930-4 28624908

[B39] Taylor J. Butler D. (2017). R Package ASMap: efficient genetic linkage map construction and diagnosis. *J. Stat. Softw.* 79 1–29. 10.18637/jss.v079.i0630220889

[B40] Tyler J. M. Webster J. A. Merkle O. G. (1987). Designations for genes in wheat germplasm conferring greenbug resistance. *Crop Sci.* 27 526–527. 10.2135/cropsci1987.0011183X002700030020x

[B41] Weng Y. Lazar M. D. (2002). Amplified fragment length polymorphism- and simple sequence repeat-based molecular tagging and mapping of greenbug resistance gene Gb3 in wheat. *Plant Breed.* 121 218–223.

[B42] Weng Y. Li W. Devkota R. N. Rudd J. C. (2005). Microsatellite markers associated with two *Aegilops tauschii*-derived greenbug resistance loci in wheat. *Theor. Appl. Genet.* 110 462–469. 10.1007/s00122-004-1853-z 15592809

[B43] Wenzl P. Carling J. Kudrna D. Jaccoud D. Huttner E. Kleinhofs A. (2004). Diversity arrays technology (DArT) for whole-genome profiling of barley. *Proc. Natl. Acad. Sci. U.S.A.* 101 9915–9920. 10.1073/pnas.0401076101 15192146 PMC470773

[B44] Woodcock B. A. Bullock J. M. Shore R. F. Heard M. S. Pereira M. G. Redhead J. (2017). Country-specific effects of neonicotinoid pesticides on honey bees and wild bees. *Science* 356 1393–1395. 10.1126/science.aaa1190 28663502

[B45] Wu Y. Bhat P. R. Close T. J. Lonardi S. (2008). Efficient and accurate construction of genetic linkage maps from the minimum spanning tree of a graph. *PLoS Genet.* 4:e1000212. 10.1371/journal.pgen.1000212 18846212 PMC2556103

[B46] Zerbino D. R. Achuthan P. Akanni W. Amode M. R. Barrell D. Bhai J. (2018). Ensembl 2018. *Nucleic Acids Res.* 46 D754–D761. 10.1093/nar/gkx1098 29155950 PMC5753206

[B47] Zhu L. C. Smith C. M. Fritz A. Boyko E. Voothuluru P. Gill B. S. (2005). Inheritance and molecular mapping of new greenbug resistance genes in wheat germplasms derived from *Alegilops tauschii*. *Theor. Appl. Genet.* 111 831–837. 10.1007/s00122-005-0003-6 16025306

[B48] Zhu L. C. Smith C. M. Fritz A. Boyko E. V. Flinn M. B. (2004). Genetic analysis and molecular mapping of a wheat gene conferring tolerance to the greenbug (*Schizaphis graminum* Rondani). *Theor. Appl. Genet.* 109 289–293. 10.1007/s00122-004-1632-x 15138689

